# Algae-derived hydrocolloids in foods: applications and health-related issues

**DOI:** 10.1080/21655979.2021.1946359

**Published:** 2021-07-19

**Authors:** Yu-Chen Liao, Chia-Che Chang, Dillirani Nagarajan, Chun-Yen Chen, Jo-Shu Chang

**Affiliations:** a Department of Chemical Engineering, National Cheng Kung University, Tainan, Taiwan; b Institute of Biomedical Sciences, National Chung Hsing University, Taichung, Taiwan; c Department of Biotechnology, Asia University, Taichung, Taiwan; d Department of Medical Research, China Medical University Hospital, Taichung, Taiwan; e Traditional Herbal Medicine Research Center, Taipei Medical University Hospital, Taipei, Taiwan; f Department of Chemical Engineering, National Taiwan University, Taipei, Taiwan; g University Center for Bioscience and Biotechnology, National Cheng Kung University, Tainan, Taiwan; h Department of Chemical and Materials Engineering, Tunghai University, Taichung, Taiwan; i Research Center for Smart Sustainable Circular Economy, Tunghai University, Taichung, Taiwan; j Research Center for Circular Economy, National Cheng Kung University, Tainan, Taiwan

**Keywords:** Algae, food additive, hydrocolloid, carrageenan, agar, alginate

## Abstract

Hydrocolloids are a class of food additives with broad applications in the food industry to develop structure in food ingredients. Hydrocolloids can be synthetic, plant-based, or animal-based. Increasing consumer awareness has led to the use of natural food ingredients derived from natural sources, making algae-derived hydrocolloids more appealing nowadays. Algae-derived hydrocolloids such as carrageenan, agar, and alginate are widely used in the food industry as thickening, gelling, and emulsifying agents. Carrageenans are sulfated polysaccharides with diverse structural specificities. The safety of carrageenan use in the food industry has been widely debated recently due to the reported pro-inflammatory activities of carrageenan and the probable digestion of carrageenan by the gut microbiota to generate pro-inflammatory oligosaccharides. In contrast, both agar and alginate are primarily nontoxic, and generally no dispute regarding the use of the same in food ingredients. This review provides an overview of the algae industry, the food additives, the algae-derived hydrocolloids, the applications of algae-derived hydrocolloids in food industries, health-related studies, and other sectors, along with future perspectives. Even though differences of opinion exist in the use of carrageenan, it is continued to be used by the food industry and will be used until suitable alternatives are available. In summary, algal hydrocolloids are ‘label-friendly’ and considered a safe option against synthetic additives.

## Graphical Abstract

**Figure:**
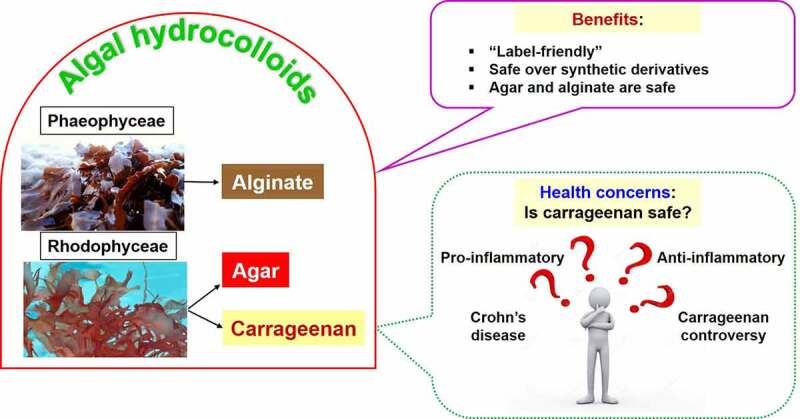

## 1. Introduction

Exploring sustainable food ingredients and promoting human health are two of the main challenges the world is currently facing. Thus, it is critical not only to feed the growing human population but also to maintain or improve their health conditions [1]. Food additives are substances that are added to food to improve the quality/consistency/shelf life of the prepared food, and the definition of a food additive varies based on State regulations. In the United States, a food additive is defined as ‘any substance the intended use of which results or may reasonably be expected to result – directly or indirectly – in its becoming a component or otherwise affecting the characteristics of any food’. This deﬁnition includes any substance used in the production, processing, treatment, packaging, transportation, or storage of food [2]. On the other hand, the Food Protection Committee of the Food and Nutrition Board defines a food additive as ‘a substance or mixture of substances, other than a basic foodstuff, which is present in a food as a result of any aspect of production, processing, storage, or packaging. The term does not include chance contaminants.’ [3]

Direct food additives are substances that are intentionally added to food products for specific functional purposes, in controlled amounts, usually at low levels (from parts per million, ppm, to 1–2% by weight). In contrast, indirect or nonintentional food additives are those entering into food products in trace amounts as a result of growing, processing, packaging, storage, or other handling [4,5]. It is mainly the quantity used in any given formulation that differentiates food additives from food ingredients. Food ingredients can usually be consumed alone as food. In contrast, food additives are primarily used in small quantities relative to the total food consumption, which nonetheless play a large part in the production of desirable and safe food products [6]. Many direct additives are revealed on the ingredient labels of food products [7].

The term ‘hydrocolloid’ indicates a heterogeneous group of high molecular weight, long-chain hydrophilic polymers (i.e. polysaccharides and proteins with polar or charged functional groups) that can perform gelling, thickening, and stabilizing functions when dispersed in water [8,9]. Most hydrocolloids are classified as food additives (e.g. food stabilizers, thickeners, and gelling agents) for controlling moisture and offering structure, viscosity, flow, stability, and eating qualities [8,10,11]. Also, hydrocolloids can be used in soups, gravies, salad dressings, sauces, and toppings as thickening agents and in jam, jelly, marmalade, restructured foods, and low sugar/calorie gels as gelling agents [11]. In short, hydrocolloids are widely used in the food industry to improve shelf-life and quality attributes [8,11]. The term ‘Food Hydrocolloids’ implies that the functionalities are obtained after mixing with water [10]. The approval for food additives and purity criteria are strictly controlled by regulations [10]. According to European food law (EU Directive 95/2/EC) [12], the definitions of stabilizers, thickeners, and gelling agents are quoted as follows: Stabilizers are substances which make it possible to maintain the physicochemical state of a foodstuff; stabilizers include substances which enable the maintenance of a homogeneous dispersion of two or more immiscible substances in a foodstuff and include also substances which stabilize, retain or intensify an existing color of a foodstuff. Thickeners are substances which increase the viscosity of a foodstuff. Gelling agents are substances which give a foodstuff texture through the formation of a gel [13].

Algae are the most abundant primary producers, and algae-derived hydrocolloids are high-value thickening, gelling, and emulsifying agents, which have reached 100,000 tons of annual global production and exceeded US$ 1.1 billion of gross market value [14,15].

The aim of this review is to compile and analyze the recent trends in the applications of algae-derived hydrocolloids in the food industry and provide an oulook regarding the safe use of the same as food additives. In this context, this review presents comprehensive information regarding common algae-derived hydrocolloids, namely carrageenan, agar, and alginate. It covers these hydrocolloids’ background, their current applications in the food industry as food additives, and their potential health impacts and controversies. Among these, the health risks associated with carrageenan consumption and the continued use of carrageenan as a food additive have been debated vigorously in recent years. Also, the so-called ‘carrageenan controversy’ is discussed in detail and critically analyzed in this review. Lastly, the updated information about algal hydrocolloids is compiled, along with future perspectives.

## 2. The ‘algae’ industry

**Figure 1.**
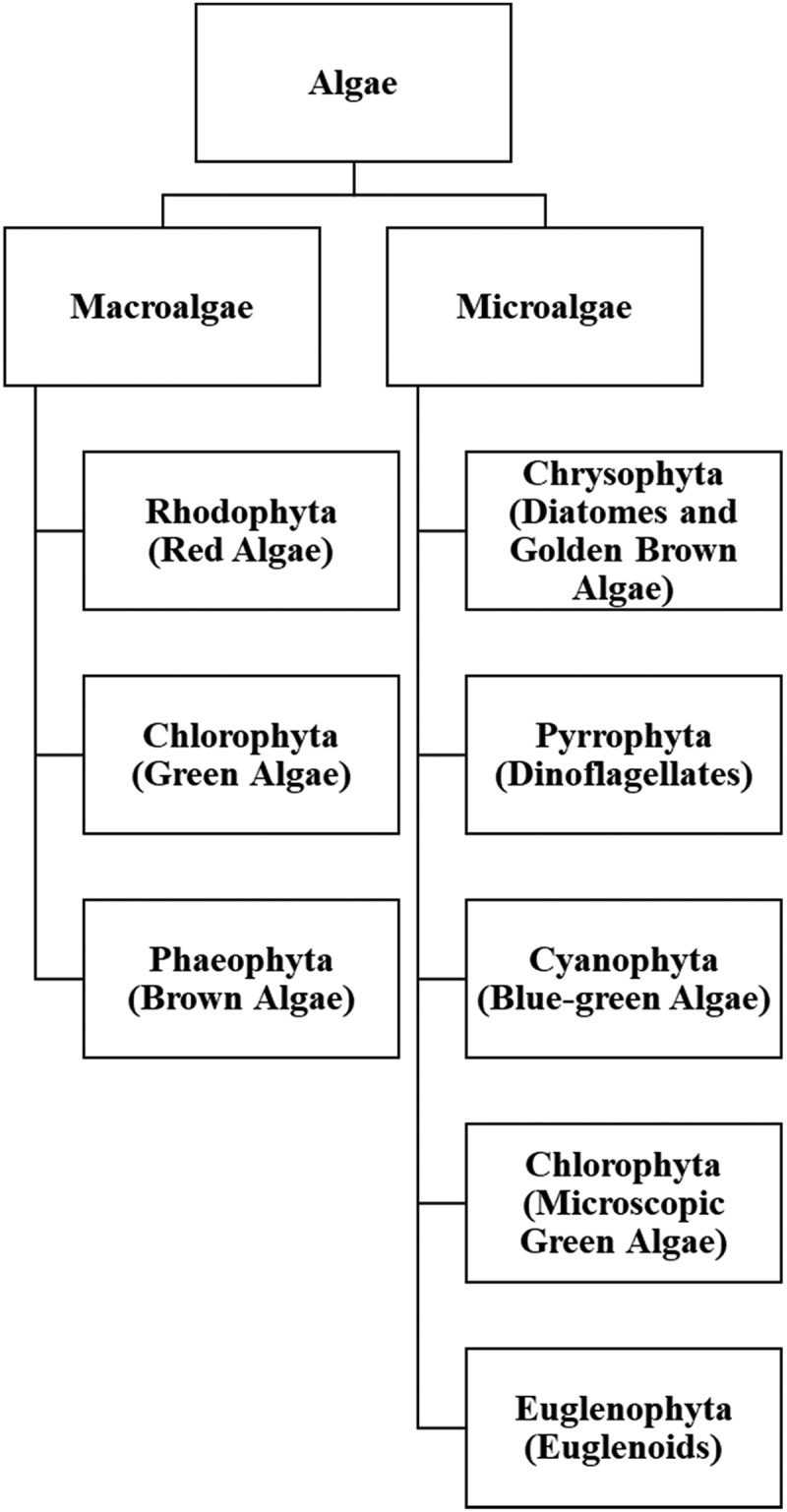
The simple classification of algae. Adapted from 110 and 111

The non-taxonomic term ‘algae’ is a highly heterogeneous group consisting of more than 40,000 species, primarily eukaryotes and typically (but not necessarily) living in aquatic habitats. The ‘algae’ includes eukaryotic phyla of Rhodophyta (red algae), Chlorophyta (green algae), Phaeophyta (brown algae), Bacillariophyta (diatoms), and dinoflagellates, as well as the prokaryotic phylum of Cyanobacteria (blue-green algae) (Figure 1) [16,17].

Algae can be classified into microalgae and macroalgae (Figure 1) [18]. Microalgae, either eukaryotes or prokaryotes, are aquatic and need to be small in size, unicellular or colonial without cell differentiation, pigment-producing, and photoautotrophic [19]. Macroalgae can be further categorized into red, brown, and green algae based on their primary pigments (Figure 1) [18]. Red and brown algae can be employed to produce hydrocolloids (e.g. agar, alginate, and carrageenan) as thickening and gelling agents [20,21]. Notably, algae are mostly autotrophs, a feature allowing simple and economical cultivation for commercial production of food additives, cosmetics, animal feed additives, pigments, polysaccharides, fatty acids, and biomass [16].

The first attempts at on-site algal cultivation started in 1731. Alginate, the main commercial phycocolloid, started gaining its industrial value in the early twentieth century [17,22]. In the 1980s, there were 46 large-scale microalgal production facilities in Asia (mainly for *Chlorella* sp.), and the large-scale production of cyanobacteria began in India. The commercial β-carotene production using halophilic green alga *Dunaliella salina* in Australia, Israel, and the United States became the third-largest microalgal industry [17,23].

In the 1990s, the photobioreactor technology has made the large-scale production of astaxanthin (which can be used in pharmaceuticals, nutraceuticals, agriculture, and animal nutrition) using *Haematococcus pluvialis* commercially viable in the United States and India [17,23,24]. Over the past few decades, we have witnessed steady growth and significant diversification in the algal biotechnology and industry [17, 23]. Algae also play a vital role in waste bioremediation and resource recovery into valuable products [25,26].

Besides used widely as food for direct human consumption, algal products are served as ingredients for biofuels, foods and food supplements, nutraceuticals, pharmaceuticals, cosmetics, fertilizers, and animal feed additives [13,27–30]. Currently, about 1 million tons of wet macroalgae are harvested and extracted for producing approximately 55,000 tons of hydrocolloids, with a total annual value of around US$ 600 million [21]. Among microalgae, *Chlorella* and *Spirulina* (*Arthrospira*) dominate the global microalgae market as popular nutraceuticals because of their high-protein content, nutritional value, and easy cultivation [31]. In addition, *Haematococcus* sp. is popular as a source of astaxanthin for the coloration of salmonid fish products [17,24,32,33].

It is noteworthy that many algal secondary metabolites, such as antioxidants, pigments, and vitamins, have been known to deliver beneficial attributes to dermaceutical products, including protection from ultraviolet (UV) radiations and prevention of photoaging such as rough skin surface, fine lines and wrinkles, uneven pigmentation, flaccidity, hair loss, and proliferative lesions [34–36]. Also, being considered safe materials from environmental resources, algal components are frequently used in cosmetic products as thickening agents and water-binding agents [17,34]. Typical algal species utilized in the cosmeceutical industry include *Chondrus crispus, Mastocarpus stellatus, Laminaria* spp., *Porphyra* spp., *Ulva lactuca, Ascophyllum nodosum, Alaria esculenta, Spirulina platensis, Nannochloropsis oculata, Chlorella vulgaris*, and *Dunaliella salina* [17,37].

## 3. Algal hydrocolloids

### 3.1. Carrageenan

Rhodophyceae, the red marine macroalgae, contains linear sulfated polysaccharides that fill the voids of its cellulose structure [11]. These polysaccharides, including carrageenan, furcellaran, and agar, all have a galactose backbone but differ in the proportion of 3,6-anhydrogalactose and the proportion and location of ester sulfate groups, leading to distinct rheological behaviors and different applications in the food industry [38]. Carrageenan is widely used in food products (especially dairy and meat products) to confer thickening, gelling, stabilizing, and strong protein-binding properties [38,39]. Most of the carrageenan-producing macroalgae are cultivated at the coastal waters of Philippines, Indonesia, and Chile [11].

**Figure 2.**
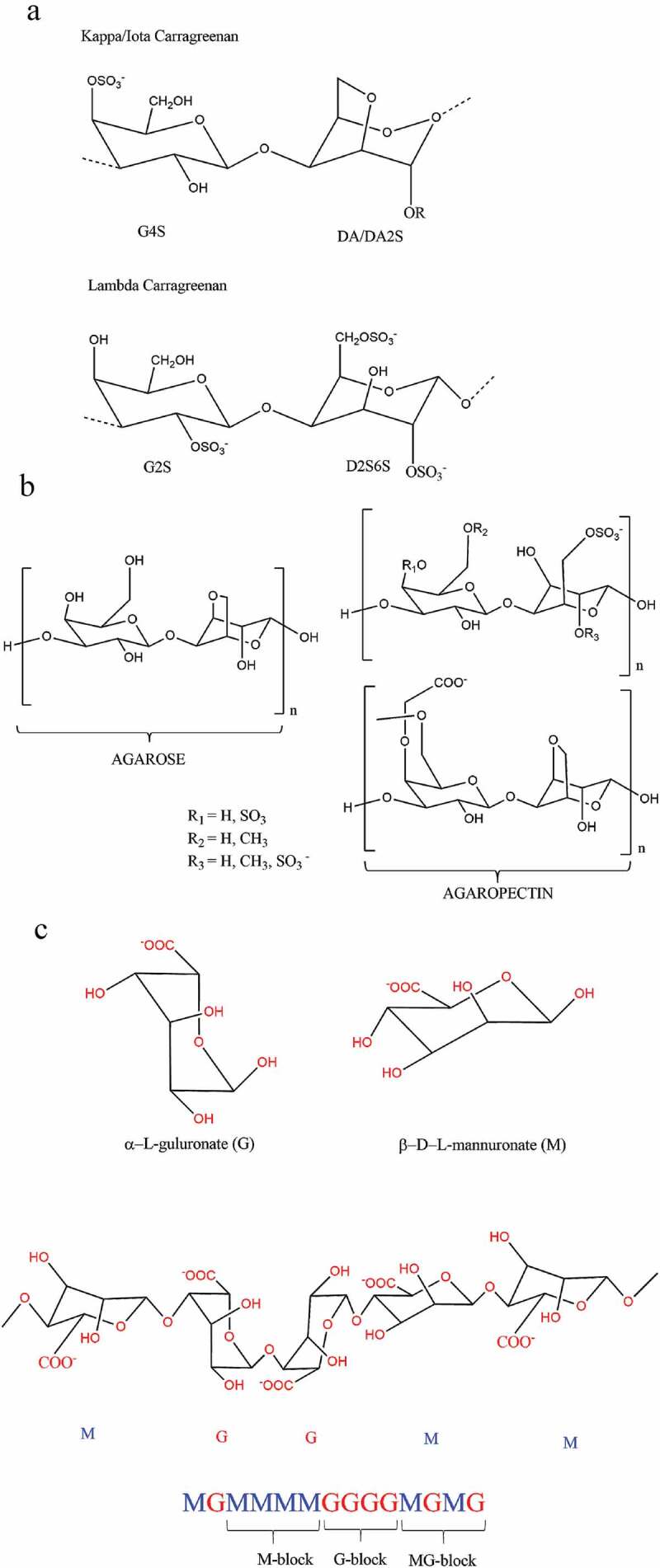
Chemical structures of algae-derived hydrocolloids. **(A]** Three main types of carrageenan, (b) Agar – agarose and agaropectin, and (c) Alginate-β-D-mannuronate (M and -L-guluronate [G). Adapted from 112,113 114, and 77

Carrageenan is a vegetarian/vegan alternative to bovine gelatin in the confectionery and pharmaceutical industries [40,41]. There are three main types of carrageenan – kappa, iota, and lambda, which differ in the degree of sulfation and thus have different gel strength, texture, solubility, melting and setting temperatures, syneresis, and synergy properties (Figure 2a) [11,39]. Kappa-carrageenan has one sulfate per disaccharide, while iota-carrageenan and lambda-carrageenan contain two and three sulfates per disaccharide, respectively (Figure 2a) [42].

Properties of the three main types of carrageenan are summarized in Table 1, and the structure is depicted in Figure 2a. It is worth noting that the properties of kappa- and iota-carrageenan are affected by the presence of salts in food products [11,21]. Kappa-carrageenan forms a strong, rigid, but brittle gel in the presence of potassium ions and can react with dairy proteins; in contrast, iota-carrageenan forms soft and elastic gel in the presence of calcium ions [11,21,38]. Lambda-carrageenan is a pure thickener and does not gel [43]. Besides, kappa-carrageenan has poor freeze-thaw stability, while iota-carrageenan shows excellent freeze-thaw stability. Accordingly, the blended kappa- and iota-carrageenan is commonly applied for desired texture, stability, and water-binding properties [11,44].

**Table 1.** Properties of the three main types of carrageenan. Adapted from 38

Carrageenan type	Kappa	Iota	Lambda
**Degree of sulfation**	One sulfate per disaccharide	Two sulfates per disaccharide	Three sulfates per disaccharide
**Solubility**			
Hot (80°C] water	Soluble	Soluble	Soluble
Cold (20°C) water	Sodium salt soluble	Sodium salt soluble	All salt soluble
Hot (80°C) milk	Soluble	Soluble	Soluble
Cold (20°C) milk	Insoluble	Insoluble	Thickens
Cold milk (TSPP added)	Thickens or gels	Thickens or gels	Increased thickening or gelling
50% sugar solutions	Soluble hot	Insoluble	Soluble
10% salt solutions	Insoluble	Soluble hot	Soluble hot
**Gelation**			
Effect of cations	Strongest gels with potassium	Strongest gels with calcium	Non-gelling
Gel texture	Firm, brittle	Soft, elastic	Non-gelling
Syneresis	Yes	No	Non-gelling
Hysteresis	10–20°C	5–10°C	Non-gelling
Freeze-thaw stability	No	Yes	Yes
Synergy with locust bean gum	Yes	No	No
Synergy with konjac glucomannan	Yes	No	No
Synergy with starch	No	Yes	No
Shear reversibility	No	Yes	Yes
Acid stability	Hydrolysis in solution, accelerated by heat; gels are stable	Hydrolysis in solution, accelerated by heat; gels are stable	Hydrolysis
Protein reactivity	Specific reaction with kappa-casein	Strong protein interaction in acid	Strong protein interaction in acid

All types of carrageenan are soluble in both hot water (80°C) and hot milk (80°C), but only lambda-carrageenan and the sodium salts of kappa- and iota-carrageenan are soluble in cold water (20°C) [11,39]. In addition, only lambda-carrageenan is soluble in cold milk and can produce a thickening effect through protein interactions, which can be enhanced by the addition of phosphates [39]. These carrageenan solutions can be used as thickeners that give a creamy, smooth, and non-gummy texture in dairy products and display pronounced pseudoplasticity (shear thinning) behavior when pumped or stirred [11].

#### 3.1.1. Carrageenan as food additives and its health-related issues

Japanese nori and algal hydrocolloids are the most consumed algal products annually, and carrageenan is the leader of algal hydrocolloids in this regard [45]. Carrageenan is an excellent alternative to emulsify salts, and it can stabilize cheese fat without altering the Ca:P ratio to produce homogeneous cheese products [46]. Also, carrageenan can be applied to stabilize the structure of cheese analogs and replace casein for imitating cheese products [46].

Carrageenan is ‘generally recognized as safe (GRAS)’ and is globally approved as a food additive, even for infant formula. Nevertheless, the ‘carrageenan controversy’ was evolved from a study reporting carrageenan’s promoting effect on inflammatory gene expression in the intestinal epithelium [45,47]. Different viewpoints in the ‘carrageenan controversy’ are summarized in Table 2. Notably, three unresolved gaps of ‘carrageenan controversy’ were highlighted in previous studies: (1) The public exposure level to carrageenan needs to be determined; (2) the differential digestive fate of carrageenan in the gastrointestinal tract has to be resolved; (3) more information is required to elucidate the impact of carrageenan on the human digestive system [47]. Thus, additional studies are warranted in the future to elucidate carrageenan’s adverse effects.

**Table 2.** Different viewpoints in ‘the Carrageenan Controversy’

**The carrageenan controversy** [45]
1. Carrageenan is approved for food use by all major food regulatory agencies worldwide.
2. Carrageenan is approved for use in liquid infant formula.
3. The negative attitude toward carrageenan is evolved from the research using an *in-vitro* model.
4. Bloggers made the issue go viral.
5. 106,found that carrageenan did not cross intestinal epithelial cell wall, was not cytotoxic, did not increase oxidative stress, and did not induce pro-inflammatory gene expression.
**Reply to comments regarding ‘the Carrageenan controversy’** [107]
1. Carrageenan has been used in labs for decades to induce inflammation.
2. Carrageenan has been used in an ever-increasing list of foods, and one may consume several grams per day, day after day for years.
3. The potential contribution of carrageenan to chronic human diseases is unknown.
4. Carrageenan is unarguably inflammatory, and its usage is a serious public issue.
5. More than 10,000 publications on PubMed website are about carrageenan, and carrageenan exposure predictably and reproducibly provokes inflammation.
6. Inflammation leads to human diseases, including atherosclerosis, cancer, arthritis, colitis, diabetes, and other diseases.
7. Carrageenan exposure may lead to relapses of ulcerative colitis and diabetes.
8. Exposure to a low dose of undegraded lambda-carrageenan may change the gene expression profiles of a human colonic epithelial cell line.
9. Food-grade carrageenan contains some low-molecular weight carrageenan.
**Revisiting the carrageenan controversy: do we really understand the digestive fate and safety of carrageenan in our foods?** [47]
1. There are gaps in our understanding of carrageenan: (1) Current levels of public exposure are unknown; (2) The link between carrageenan physicochemical properties and the impact on digestive proteolysis, microbiome, and inflammation are not yet resolved; (3) The digestive fate of carrageenan and the predisposed populations need to be determined.
2. Carrageenan is approved for food use by all major food regulatory agencies worldwide, but the supportive evidence may either based on animal studies or studies that are ethically challenged.
3. Carrageenan toxicology and possible health impacts have been reported in extensive *in vivo* and *in-vitro* studies since the 1970s.
4. Carrageenan may cause neoplasms, tumors, and ulcers in the small intestine and colon.
5. Carrageenan can play a role in the onset of glucose intolerance in mice.
6. Carrageenan may lead to an earlier relapse in ulcerative colitis in humans.
7. Low doses of undegraded carrageenan can activate inflammatory pathways *in-vitro*.
8. The low-MW (degraded) carrageenan can undisputedly provoke epithelial ulcerations and induce secretion of inflammatory cytokines.
9. Low-MW carrageenan diffuses through the mucus layer and to the intestinal epithelium faster.
10. The risk from the hypothesized physiologically digested carrageenan needs to be confirmed.
**Comment on ‘Revisiting the carrageenan controversy: do we really understand the digestive fate and safety of carrageenan in our foods?’** [108]
1. Poligeenan or degraded carrageenan used for testing are generated under very harsh conditions and are not the same as commercial carrageenan.
2. High-MW carrageenan is a food additive and is never intentionally injected into humans to induce significant inflammatory responses.
3. If food-grade carrageenan is stable in the gut, it cannot induce inflammatory responses.
4. No allergy or anaphylaxis caused by carrageenan is reported in humans.
5. Well-designed studies with good laboratory practices showed no harmful effects of dietary carrageenan.
6. The carcinogenic effect of carrageenan needs to be confirmed with long-term animal studies.
7. No adverse effects of carrageenan were observed in recent clinical trials.
8. The *in-vitro* adverse effects of carrageenan cannot be reproduced.
9. There is no controversy, only confusion.
10. Carrageenan does not cause inflammatory or gastrointestinal effects in animals using standard protocols for safety evaluation.
11. Degraded carrageenan (Poligeenan) is not permitted to be used as a food additive.
12. Carrageenan injection is not relevant to the usage as a food additive.
**Reply to the Comment on ‘Revisiting the carrageenan controversy: do we really understand the digestive fate and safety of carrageenan in our foods?’** [109]
1. There is little information on (1) the physicochemical properties of commercial carrageenan, (2) carrageenan exposure levels in human diets, (3) the role of carrageenan in gut microbiome dysbiosis and inflammation, and [4) the effects of carrageenan on susceptible populations.
2. Food-grade carrageenan has possible adverse effects on digestive proteolysis, epithelial integrity, and human health in double-blind, placebo-controlled independent trials.
3. Carrageenan is a complicated mixture of natural biopolymers, thus posing significant challenges for analyzing its physicochemical characteristics.
4. The molecular-weight distributions of carrageenan are not disclosed.
5. There is no confusion over carrageenan terminology.
6. Studies for enhancing our understanding of carrageenan’s broad-spectrum, polydisperse nature, and possible bio-transformations during human digestion are warranted.
7. The challenge of experimental reproducibility of carrageenan will be overcome by independent trials.
8. Possible impacts of long-term carrageenan exposure on the gut ecosystem and low-grade inflammation should be determined.

The daily level of carrageenan consumption in the United States and France was only 45–100 mg, but it has increased significantly since the 1980s. It has been estimated that human carrageenan intake has elevated to 2.5 g per day in 1989 and up to 7.7 g per day (0–1 mg/kg body weight) in south Florida in 2003 [47,48]. Still, health agencies have not yet revisited carrageenan intake levels nor specifying the acceptable daily intake (ADI) of carrageenan [47,49].

Carrageenan is generally classified as an indigestible dietary carbohydrate, but some pieces of evidence supported that kappa-carrageenan could depolymerize in gastric juices and result in degraded carrageenan (molecular weight (MW) <100 kDa) [47,50]. Several studies also revealed that carrageenan might inhibit human gastric juices and interfere with digestive proteolysis, leading to anti-nutritional effects [47,51–53]. For instance, food-grade *iota*-carrageenan engaged in electrostatic bonding with lactoferrin nano-particles, resulting in a charged compound with a zeta potential value of −69.2 mV, and this compound could resist gastric digestion of lactoferrin up to 1 h [51]. Gel formation by *kappa-*carrageenan at low concentrations was delayed by 20 minutes in the presence of cow’s milk (with 9% total solids), while 0.1% carrageenan formed gel within 20 minutes at 20°C [52]. Since low-MW carrageenan (i.e. <100 kDa) is regarded as an established food contaminant and few reports are available concerning the digestive proteolysis of carrageenan, further studies are required to clarify carrageenan’s digestive fate and its resultant impact on human health [47].

Although being disputed, several *in-vitro* studies indicated that carrageenan can elicit inflammation by engaging toll-like receptor (TLR)4/B-cell leukemia/lymphoma (BCL)10-dependent activation of nuclear factor kappa-light-chain-enhancer of activated B cells (NF-κB), which upregulates Interleukin-8 (IL-8) to trigger inflammatory reactions. These studies revealed that, in human colinic epithelial cell line NCM640, low-dose of undegraded lambda-carrageenan (1 μg/ml) treatment led to an increase in Bcl10, a crucial innate immunity mediator, which induces NF-κB activation. In turn, the activated NF-κB promotes the expression of IL-8 to trigger inflammation but also upregulates Bcl10 to sustain NF-κB activation [54,55]. Additionally, the lambda-carrageenan-induced increase of Bcl10 and IL-8 depends on TLR4, an innate immunity receptor, as lambda-carrageenan failed to upregulate Bcl10 and IL-8 in both NCM640 cells and mouse macrophage cell line RAW 264.7 when their TLR4 were functionally blocked by TLR4 antibody, nor in TLR4-deficient mouse macrophage cell line 23ScCr [54]. Moreover, low-MW carrageenan (or degraded carrageenan) with higher epithelial diffusion rates has been demonstrated as the cause of adverse effects, which might elicit lysosomal disruption in macrophages to provoke epithelial ulcerations but also induce monocytes to produce pro-inflammatory molecules, such as tumor necrosis factor α (TNF-α) and intercellular adhesion molecule 1 (ICAM-1) [47,48,56]. Intriguingly, the gut microbiota has also been implicated to play an active role in carrageenan’s metabolism. Although carrageenan is expected inert to hydrolysis by intestinal enzymes, some colonic bacteria can de-sulfate carrageenan [47,57–59]. *In-vitro* fermentation study further revealed that kappa-carrageenan oligosaccharides (KO3 and KO6) obtained from simulated gastric digestion were gradually degraded and utilized by gut microbes, which in turn modulate gut microbiota composition and the production of short-chain fatty acids by gut microbes [60].

Additional evidence has substantiated the pro-inflammatory effect of carrageenan. The kappa-carrageenan oligosaccharides KO3 and KO6 have been shown to promote pro-inflammatory bacteria Prevotella while suppressing anti-inflammatory bacteria Bacteroides and Parabacteroides. Moreover, the smaller kappa-carrageenan oligosaccharides might provoke human colorectal cancer cell line HT-29 to secret pro-inflammatory cytokines (IL-1β, TNF-α) in addition to secretory immunoglobulin A (SIgA) and mucin 2 (human mucus gel forming protein) [60].

In addition to pro-inflammatory effects, carrageenan could exert anti-inflammatory action. Carrageenan is commonly used as a ‘pro-inflammatory agent’ for developing pleurisy, intestinal inflammation, paw edema, prostatitis, colitis, and arthritis in animal models [60–64]. Nevertheless, kappa-carrageenan hexamer, the negatively charged sulfated oligosaccharides, has been shown to suppress lipopolysaccharide (LPS)-elicited pro-inflammatory responses in murine macrophage cell RAW264.7 *via* inhibiting the cluster of differentiation 14 (CD14)/REL-dependent NF-κB inflammatory pathway [65].

It is noteworthy that a recent study revealed children with Crohn’s Disease, an inflammatory colon disorder, frequently consume particular food additives, such as aluminosilicates, carboxymethylcellulose, carrageenan, maltodextrin, polysorbate-80, soy lecithin, titanium dioxide, and xanthan gum, although the impacts of these additives on the pathogenesis of Crohn’s Disease remain verified [66]. On the contrary, the iota-carrageenan produced from red macroalgae *Sarconema filiforme* was reported to attenuate high-carbohydrate, high-fat diet-induced metabolic syndromes (obesity, hypertension, dyslipidaemia, glucose intolerance, fatty liver, and increased left ventricular collagen deposition) and modulate gut microbiota in male Wistar rats [67].

Carrageenan-elicited pro-inflammatory response has raised a significant health concern on using carrageenan as food additives. In November 2016, the National Organics Standards Board (NOSB), which advises the US Department of Agriculture (USDA) on organic products-related issues, voted 10 to 4 to remove carrageenan from the National List of additives approved for use in foods labeled ‘USDA Organic’ [45,68]. Still, USDA did not take the advice, and carrageenan is still on the ‘Generally Recognized as Safe (GRAS)’ food additives list of the US Food and Drug Administration (FDA) after the review in April 2018 [68]. Thus, it is necessary to conduct extensive human trials and epidemiological studies to resolve the health-related issue of the ‘carrageenan controversy’ for future application of carrageenan in the food industry.

### 3.2. Agar

Agar (or agar–agar) is a hydrocolloid (or phycocolloid) extracted from red macroalgae (Rhodophyceae). It has been used as a gelling, thickening, and stabilizing food additive for more than 350 years since discovered in Japan in 1658 [69,70]. Agar is composed of two types of polysaccharides, namely, agarose (a linear polysaccharide) and a heterogeneous mixture of smaller molecules called agaropectin (Figure 2b). Notably, agarose is the main gelling agent in agar [69,71].

Agar is insoluble in cold water, while colloidally dispersible in hot water (>90°C). When cooled at 32–39°C, a 1.5% agar solution will form a firm and brittle gel that does not melt by heating below 85°C [70,71]. Agar is a very efficient gelling agent, which can form a firm, brittle, and thermally reversible gel at low concentration (0.2%) [71]. Agar gel is formed by hydrogen bonds between the adjacent D-galactose and 3,6-anhydro-L-galactose along the linear chains of agarose with repeating units. In this context, no additional agent is required to form an agar gel, and the gel structure is not affected by salts or proteins [71].

Agar is commonly used in microbiology research for preparing solid culture media. Also, agar is widely used in culinary, food, and confectionery industries as the gelling agent for producing Asian traditional dishes, canned meats, confectionery jellies, and aerated products like marshmallows, nougat, and toffees [71]. Comparing to gelatin, the higher melting point of agar gel makes products more thermostable, which is especially useful for preparing bakery fillings [71].

#### 3.2.1. Agar as food additives and its health-related issues

Agar is a widely used food additive, and it has been approved by the US Food and Drug Administration (FDA) as ‘Generally Recognized as Safe (GRAS)’ since FDA started classifying food additives in 1972. It is noteworthy that no adverse effect of agar on humans has ever been reported during its long-lasting utilization history [72]. In Asia, agar is a common food additive, and it is commonly used to make foods that require heating (e.g. fry, bake, broil, roast, toast, and barbecue) before consumption, such as cake, sausage, roast pork, and bacon [73]. Agar fluid gels can also be used for producing very stable foams to replace fat in whipped products [74].

Agar can be thermally degraded when the heating temperature is above 250°C [73]. Thermal degradation of agar was found to be a single-step exothermic reaction, and the degradation temperature is positively correlated with gel strength [73]. Although mostly nontoxic, some thermal-degraded agar products, such as furyl hydroxymethyl ketone, furfural, and 5-(hydroxymethyl)-2-furancarboxaldehyde (HFM), can exert some toxicity to humans [73]. The IC_50_ of furan for mice is 120 mg/m^3^, while some stimulatory and anesthetic effects have been reported in humans [73].

### 3.3. Alginate

Alginates are gel-forming hydrophilic polysaccharides in the cell walls of a wide range of brown macroalgae found on the coasts of the North Atlantic, South America, and Asia [75]. Alginates are polymers of mannuronate (M) and guluronate (G) covalently linked together in different blocks, including blocks of consecutive G residues called G blocks, consecutive M residues called M blocks, and alternating M and G residues called MG blocks (Figure 2c) [11]. Similar to agar, alginates are commonly used for gelling, thickening, stabilizing, and film-forming applications. Alginates are present as a mixed salt of sodium and/or potassium, calcium, and magnesium in brown macroalgae, but only sodium alginate is predominantly used in foods [11,75,76]. Alginates derived from different brown macroalgae display slightly different structures, leading to different gelling properties [11].

Unlike other hydrocolloids, alginates are unique in their cold solubility, which allows the production of cold-setting gels, heat/temperature-independent non-melting gels, and freeze-thaw stable gels [76]. Notably, the addition of cations such as calcium is required for alginate gel formation. Only G blocks and sometimes MG blocks can react with calcium to form alginate gels [77]. Thus, the higher the G residues, the stronger the alginate gels [11,75,78]. It should be aware that, since alginates are very reactive to calcium, the release of calcium must be carefully adjusted to prevent pre-gel formation called lumps or ‘fish eyes’. Also, controlling the alginates-calcium interaction would confer shear-irreversible and heat-stable properties on cold-setting gels [11,75]. The controlled conditions can be accomplished by using suitable calcium sequestrants such as citrate or phosphate [77] or by processing at temperatures above 70°C and setting by cooling [75].

The internal setting under controlled conditions is obligatory for most alginate applications, including bakery fillings, custards, structured fruits, structured vegetables, structured meat products, and aerated confectioneries [11]. For bakery applications, alginate gels are stable at baking temperatures and various sugar levels for making heat-stable bakery and fruit fillings, and their cold-setting properties allow the production of instant bakery filling creams [11,75]. In addition, sodium alginate can be used as the thickening and structuring agent in low-fat margarine and spread products, and also used for controlling the melting behavior of ice cream [11]. Other alginate applications in food products include reformed foods such as onion rings and olive fillings [75].

#### 3.3.1. Alginate as food additives and its health-related issues

Bovine serum albumin (BSA) is a common food protein ingredient found in bovine whey and blood. Given its physicochemical and structural properties have been well studied, BSA serves as a good research model for protein interaction in foods [79]. The interaction between BSA and sodium alginate demonstrated that alginate hardly induces changes in BSA’s secondary structure but instead acting as a stabilizer to increase BSA protein stability [79].

Gum Arabic/gelatin/alginate microcapsules loaded with fucoxanthin were shown resistant to simulated gastrointestinal digestion using 0.32% pepsin and 0.02% NaCl at pH 1.2 for 2 h, followed by incubation with simulated intestinal fluid with 1% trypsin, pH 7.4 for 4 h. Alginate hydrogel can be used for novel fucoxanthin encapsulation, which can improve gastric acid tolerance and the rapid disintegration and release of fucoxanthin in the small intestine, providing a good oral delivery system for fucoxanthin [80]. Also, alginate-based encapsulation can increase the survivability of *Lactobacillus plantarum* during storage and under simulated food processing and gastrointestinal conditions [81]. Alginate-chitosan and alginate-skimmed milk encapsulated *L. plantarum* showed survivability in the presence of simulated gastric fluid (pepsin 3 g/L, pH 1.2) and stimulated intestinal fluid (3 g/L bile salt, 10 g/L pancreatin, pH 7.5), respectively for up to 120 mins [81]. Likewise, microcapsules of alginate-whey protein isolate can improve the survivability and release behavior of the probiotic bacterium *Lactobacillus acidophilus* under simulated gastric and intestinal juice [82].

Minimally processed fruits can be an alternative to dairy products for probiotic delivery. It has been demonstrated that edible alginate coating can sustain the incorporated probiotic *Lactobacillus rhamnosus* CECT 8361. Similarly, the blueberries encapsulated by edible alginate coating maintain their sensory and quality attributes for up to 14 days of refrigerated storage, allowing further implementation of functional fruit products [83]. Moreover, the stability of sodium alginate-coated soybean oil body emulsions was proved to be markedly enhanced against NaCl (0–250 mM at pH 7) and freeze-thaw cycling, a discovery encouraging the application of alginate coating to the development of natural oil body-based products in the food industry [84].

For applications other than the food industry, the formulations of sodium alginate and gelatin mixtures were commonly used in bio-printing and bio-fabrication studies and applications. It has been proved that the food matrix of soy protein isolate, sodium alginate, and gelatin is a promising material in 3D food printing [85,86]. Sodium alginate was also widely used as bio-inks for 3D bio-printing [87,88]. Moreover, alginate-encapsulated lipid emulsion beads were shown to reduce food intake by overweight adults in the human trial. Hence, it is promising to apply alginate-encapsulated lipid emulsion beads to the development of easy-to-use weight management products [89].

Sodium alginates are known to be biocompatible, biodegradable, and ‘Generally Recognized as Safe (GRAS)’ with no reports about serious adverse effects of alginates as food additives [90]. Toxicology studies on sodium alginate were carried out very early in the 1940s using lab animals such as mice, rabbits, and even cats [91,92]. Although adverse effects were reported in these early studies, the intraperitoneal and intravenous delivery of high dosage (up to 250 mg/kg) of sodium alginate in those studies is far beyond the amounts of human dietary intake [91,92].

In an *in-vitro* study using a murine macrophage‐like cell line RAW264.7, sodium alginate was found to provoke inflammation through the NF‐κB pathway, accompanied by a dose (1 and 3 mg/ml alginate) and time-dependent production of pro-inflammatory cytokines, such as IL‐1β, IL‐6, IL‐12, and TNF‐α [93]. The maximum concentration of IL‐1β, IL‐6, IL‐12, and TNF‐α at 120 h was 20 pg/ml, 3 ng/ml, 20 pg/ml, and 2 ng/ml, respectively. This finding appears to indicate that sodium alginate is pro-inflammatory. In contrast, a recent *in vivo* study revealed that purified alginate could suppress the inflammatory responses elicited by non-purified alginate. This notion was evidenced by a more than 30% decrease of inflammatory cell levels and a threefold reduction in fibrotic wall thickness in Wister rats implanted with purified alginate microcapsules than its non-purified counterpart [94]. Likewise, in C57BL/6 J mice fed with a high-fat diet, alginate oligosaccharide provided at a concentration of 5 g/100 g mice feed was shown to lower the inflammation markers (i.e. IL-1β and CD-11 c) by enhancing the probiotic gut microbiota population. In this context, alginate supplementation decreased the pro-inflammatory gut bacteria *Streptococcaceae, Rikenellaceae*, and *Lachnospiraceae and Erysipelotrichaceae* by 96%, 76%, and 83%, respectively, along with a 32-fold reduction in *Erysipelotrichaceae* [95]. Conversely, alginate supplementation enriched the beneficial probiotic bacteria including *Bacteroides acidifaciens, Lactobacillus gasseri, Lactobacillus reuteri, Akkermansia muciniphila, L. reuteri*, and *L. gasseri* [95]. Also, alginate was demonstrated to mitigate acetaminophen-induced acute liver injury in mice *via* suppressing inflammation, as evidenced by the decrease in serum IL-6 levels after oral administration of alginate [96]. Thus, it appears that the findings of most health-related studies render alginates a positive public image in contrast to carrageenan.

## 4. Summary and future perspectives

Algae are the leading primary producers on earth, and algae-derived hydrocolloids, including carrageenan, agar, and alginate, are food additives extensively used in the food industry. This review gives a concise overview of the broad applications of algal hydrocolloids along with their health-related issues. The progressive trends in the novel applications of algal hydrocolloids underscore consumers’ preference for natural food additives over synthetic food additives.

Aside from the reported adverse effects and controversies as food additives, carrageenan is a promising renewable biomaterial (e.g. films and coatings) that could be an alternative to the petroleum-derived plastics used in pharmaceutical and biomedical applications [97]. Carrageenan also possesses anti-thrombotic, anti-viral, anti-cancer, and immunomodulatory properties. Moreover, carrageenan-based controlled drug delivery systems and carrageenan-derived hydrogels for 3D printing have great application and market potentials [98]. Therefore, the ‘carrageenan controversy’ will never be settled before a cheaper and better substitute appearing in the market.

Agar has excellent rheological properties and exhibits anti-coagulant, anti-viral, anti-oxidative, anti-cancer, and immunomodulatory activities. Without question, agar will continue to be widely used in food, cosmetic, pharmaceutical, biomedical, and biotechnology industries [99, W. K.^100^]. Intriguingly, agar gels are also used to clean delicate artwork surfaces. It is expected that more applications of agar will be developed in different fields due to its extreme versatility [101].

In addition to applications in the food industry, alginate-based composites can be used to remove various pollutants such as dyes, heavy metals, and antibiotics in water and wastewater, due to their biocompatible, nontoxic, and cost-effective properties [B.^102^]. Notably, alginate remains an attractive material for biomedical applications; it is promising to develop alginate microparticles as drug delivery systems for oral administration [103]. Alginate-based materials can also be used for tissue engineering and wound dressing applications [104,105]. More applications of alginates in different fields are expected to be explored in the future.

It is noteworthy that the commercial future of any food ingredient, including hydrocolloids, is determined by consumers’ perception but not by scientific facts [10]. Macroalgal extracts like carrageenan, agar, and alginates are usually ‘label-friendly’ in terms of perception and label image [10]. Although there are ‘controversies’ and limitations in the application of ‘natural’ food additives, they are still considered as a ‘safer’ option than synthetic food additives. Thus, more studies are required to either resolve the ‘controversies’ or develop new alternatives with better properties and more friendly prices.

## 5. Conclusions

Carrageenan-induced inflammatory responses have ignited controversy and rigorous debate over the safety of using carrageenan as food additives. Accumulating evidence suggests that carrageenan is intricately connected to inflammatory responses, either pro- or anti-inflammatory. Furthermore, the public exposure levels and the acceptable daily intakes (ADI) of carrageenan remain specified. Moreover, the digestive fate of carrageenan with its impacts on human health requires more studies to determine. For both agar and alginate, there is no controversy or debate on safety issues. Notably, all algae-derived hydrocolloids are ‘label-friendly’ compared to synthetic food additives, and more innovative applications of these hydrocolloids are being configured in the food and biomedical industries.

## Highlights

- Major algae-derived hydrocolloids as food additives are presented
- Algal hydrocolloids are considered as the ‘vegan’ alternative to animal products
- The unsettled carrageenan controversy related to inflammation is visited
- Both agar and alginate are safe for human consumption based on safety-related studies
- Macroalgal hydrocolloids are considered ‘label-friendly’, and are regarded safer than synthetic additives.

## References

[1] Ibañez E, Cifuentes A. Benefits of using algae as natural sources of functional ingredients. J Sci Food Agric. 2013;93(4):703–709. 23339029 10.1002/jsfa.6023

[2] Agulló E, Rodríguez MS, Ramos V, et al. Present and future role of chitin and chitosan in food. Macromol Biosci. 2003;3(10):521–530.

[3] Branen AL, Haggerty RJ. Introduction to food additive. In: Food Additives. Second ed. Marcel Dekker, Inc; 1999. p. 1–10. New York.

[4] Chia WY, Kok H, Chew KW, et al. Can algae contribute to the war with Covid-19? Bioengineered. 2021;12(1):1226–1237. 33858291 10.1080/21655979.2021.1910432 PMC8806238

[5] Considine GD, Ed. Van Nostrand’s Scientific Encyclopedia. Van Nostrand’s Scientific Encyclopedia. Hoboken, NJ, USA: John Wiley & Sons, Inc; 2005.

[6] Somogyi LP. Food Additives. In: *Kirk-Othmer Encyclopedia of Chemical Technology* (pp. 1–59). Hoboken, NJ, USA: John Wiley & Sons, Inc; 2015.

[7] Nweze CC, Mustapha AA, Olose M. Aspartame food additive and its biochemical implication: a review. Food Science and Quality Management. 2015;36:16–23.

[8] Saha D, Bhattacharya S. Hydrocolloids as thickening and gelling agents in food: a critical review. J Food Sci Technol. 2010;47(6):587–597. 23572691 10.1007/s13197-010-0162-6 PMC3551143

[9] Williams PA, Phillips GO Introduction to food hydrocolloids. In: Handbook of Hydrocolloids: second Edition. Elsevier Inc; 2009. p. 1–22.

[10] Imeson A. Food Stabilisers, Thickeners and Gelling Agents. In: Imeson A, editor. Food Stabilisers, Thickeners and Gelling Agents. Oxford, UK: Wiley-Blackwell; 2009c. p. 50–72.

[11] Pegg AM The application of natural hydrocolloids to foods and beverages. In: Baines D, Seal R, editors. Natural Food Additives, Ingredients and Flavourings. Cambridge: Elsevier; 2012. p. 175–196.

[12] EC. European Parliament and Council Directive No 95/2/EC of 20 February 1995 on food additives other than colours and sweeteners. Off J Eur Union. 1995; L0002: 1–53.

[13] Koyande AK, Chew KW, Rambabu K, et al. Microalgae: a potential alternative to health supplementation for humans. Food Sci Hum Wellness. 2019;8(1):16–24.

[14] Kovač D, Simeunović J, Babić O, et al. Algae in food and feed. Food and Feed Research, 2013;40(1):21–31.

[15] Rhein-Knudsen N, Ale MT, Meyer AS. Seaweed hydrocolloid production: an update on enzyme assisted extraction and modification technologies. Mar Drugs. 2015;13(6):3340–3359. 26023840 10.3390/md13063340 PMC4483632

[16] Gangl D, Zedler JAZ, Rajakumar PD, et al. Biotechnological exploitation of microalgae. J Exp Bot. 2015;66(22):6975–6990. . 26400987 10.1093/jxb/erv426

[17] Hallmann A. Algal transgenics and biotechnology. *Transgenic Plant J*, 2007;1(1):81–98.

[18] Hong IK, Jeon H, Lee SB. Comparison of red, brown and green seaweeds on enzymatic saccharification process. J Ind Eng Chem. 2014;20(5):2687–2691.

[19] Olaizola M. Commercial development of microalgal biotechnology: from the test tube to the marketplace. Biomol Eng. 2003;20(4–6):459–466. 12919832 10.1016/s1389-0344(03)00076-5

[20] Leong HY, Chang CK, Lim JW, et al. Liquid Biphasic Systems for Oil-Rich Algae Bioproducts Processing. Sustainability. 2019;11:4682.

[21] McHugh DJ (2003). A guide to the seaweed industry. Retrieved October8, 2020, from http://www.fao.org/3/y4765e/y4765e00.htm#Contents

[22] Pulz O, Gross W. Valuable products from biotechnology of microalgae. Appl Microbiol Biotechnol. 2004;65(6):635–648. 15300417 10.1007/s00253-004-1647-x

[23] Spolaore P, Joannis-Cassan C, Duran E, et al. Commercial applications of microalgae. J Biosci Bioeng. 2006;101(2):87–96. 16569602 10.1263/jbb.101.87

[24] Khoo KS, Lee SY, Ooi CW, et al. Recent advances in biorefinery of astaxanthin from Haematococcus pluvialis. Bioresour Technol. 2019;288:121606. 31178260 10.1016/j.biortech.2019.121606

[25] Abdul-Latif NS, Ong MY, Nomanbhay S, et al. Estimation of carbon dioxide (CO2) reduction by utilization of algal biomass bioplastic in Malaysia using carbon emission pinch analysis (CEPA). Bioengineered. 2020;11((1):):154–164. 32013677 10.1080/21655979.2020.1718471 PMC6999637

[26] Cheah WY, Show PL, Yap YJ, et al. Enhancing microalga Chlorella sorokiniana CY-1 biomass and lipid production in palm oil mill effluent (POME) using novel-designed photobioreactor. Bioengineered. 2020;11(1):61–69. 31884878 10.1080/21655979.2019.1704536 PMC6961591

[27] Chew KW, Yap JY, Show PL, et al. Microalgae biorefinery: high value products perspectives. Bioresour Technol. 2017;229:53–62. 28107722 10.1016/j.biortech.2017.01.006

[28] Cuellar‐Bermudez SP, Aguilar‐Hernandez I, Cardenas‐Chavez DL, et al. Extraction and purification of high-value metabolites from microalgae: essential lipids, astaxanthin and phycobiliproteins. Microb Biotechnol. 2015;8(2):190–209. 25223877 10.1111/1751-7915.12167 PMC4353334

[29] Michalak I, Chojnacka K, Saeid A. Plant growth biostimulants, dietary feed supplements and cosmetics formulated with supercritical CO_2_ algal extracts. Molecules. 2017;22:1. 10.3390/molecules22010066 PMC6155630 28054954

[30] Yanagisawa M, Kawai S, Murata K. Strategies for the production of high concentrations of bioethanol from seaweeds: production of high concentrations of bioethanol from seaweeds. Bioengineered. 2013;4(4):224–235. 23314751 10.4161/bioe.23396 PMC3728193

[31] Koyande AK, Show P-L, Guo R, et al. Bio-processing of algal bio-refinery: a review on current advances and future perspectives. Bioengineered. 2019;10(1):574–592. 31668124 10.1080/21655979.2019.1679697 PMC6844430

[32] García JL, deVicente M, Galán B. Microalgae, old sustainable food and fashion nutraceuticals. Microb Biotechnol. 2017;10(5):1017–1024. 28809450 10.1111/1751-7915.12800 PMC5609256

[33] Rumin J, Nicolau E, deOliveira RG, et al. Analysis of scientific research driving microalgae market opportunities in Europe. Mar Drugs. 2020;18(5):264. 10.3390/md18050264 PMC7281102 32443631

[34] Ariede MB, Candido TM, Jacome ALM, et al. Cosmetic attributes of algae - A review. Algal Res. 2017;25:483–487.

[35] Callaghan TM, Wilhelm KP. A review of ageing and an examination of clinical methods in the assessment of ageing skin. Part 2: clinical perspectives and clinical methods in the evaluation of ageing skin. Int J Cosmet Sci. 2008;30(5):323–332. 18822037 10.1111/j.1468-2494.2008.00455.x

[36] Sathasivam R, Radhakrishnan R, Hashem A, et al. Microalgae metabolites: a rich source for food and medicine. Saudi J Biol Sci. 2019;26(4):709–722. 31048995 10.1016/j.sjbs.2017.11.003 PMC6486502

[37] Munir N, Sharif N, Naz S, et al. Algae: a potent antioxidant source. Sky J Microbiol Res 2013;1(3):22–31.

[38] Imeson A Carrageenan and furcellaran. In: Phillips G, Williams P, editors. Handbook of Hydrocolloids: second Edition. Cambridge: Elsevier Inc; 2009b. p. 164–185.

[39] Blakemore WR, Harpell AR Carrageenan. In: Alan I, editor. Food Stabilisers, Thickeners and Gelling Agents. Hoboken: Wiley Online Library; 2010. p. 73–94.

[40] Campbell R, Hotchkiss S. Carrageenan industry market overview. In: Hurtado, AQ, Critchley, AT, Neish, IC, editors. Tropical Seaweed Farming Trends, Problems and Opportunities. Vol. 9. New York: Springer International Publishing; 2017. p. 193–205.

[41] Oladzadabbasabadi N, Ebadi S, Mohammadi Nafchi A, et al. Functional properties of dually modified sago starch/κ-carrageenan films: an alternative to gelatin in pharmaceutical capsules. Carbohydr Polym. 2017;160:43–51. 28115099 10.1016/j.carbpol.2016.12.042

[42] Sudhakar YN, Selvakumar M, Bhat DK. Chapter 4-Biopolymer Electrolytes for Solar Cells and Electrochemical Cells. In: Sudhakar YN, Selvakumar M, Bhat DK, editors. Biopolymer Electrolytes. Amsterdam: Elsevier; 2018. p. 117–149.

[43] Langendorff V. Effects of carrageenan type on the behaviour of carrageenan/milk mixtures. Food Hydrocoll. 2000;14(4):273–280.

[44] Yuan C, Du L, Zhang G, et al. Influence of cyclodextrins on texture behavior and freeze-thaw stability of kappa-carrageenan gel. Food Chem. 2016;210:600–605. 27211687 10.1016/j.foodchem.2016.05.014

[45] Bixler HJ. The carrageenan controversy. J Appl Phycol. 2017;29(5):2201–2207.

[46] Błaszak B, Gozdecka G, Shyichuk A. Carrageenan as a functional additive in the production of cheese and cheese-like products. Acta Scientiarum Polonorum, Technologia Alimentaria 2018;17(2):107–116. 29803212 10.17306/J.AFS.0550

[47] David S, Shani Levi C, Fahoum L, et al. Revisiting the carrageenan controversy: do we really understand the digestive fate and safety of carrageenan in our foods? Food Function. 2018;9(3):1344–1352. 29469913 10.1039/c7fo01721a

[48] Tobacman JK. Review of harmful gastrointestinal effects of carrageenan in animal experiments. Environ Health Perspect. 2001;109(10):983–994. 11675262 10.1289/ehp.01109983 PMC1242073

[49] David S, Wojciechowska A, Portmann R, et al. The impact of food-grade carrageenans and consumer age on the *in vitro* proteolysis of whey proteins. Food Res Int. 2020;130:108964. 32156399 10.1016/j.foodres.2019.108964

[50] Capron I, Yvon M, Muller G. In-vitro gastric stability of carrageenan. Food Hydrocoll. 1996;10(2):239–244.

[51] David-Birman T, Mackie A, Lesmes U. Impact of dietary fibers on the properties and proteolytic digestibility of lactoferrin nano-particles. Food Hydrocoll. 2013;31(1):33–41.

[52] Drohan DD, Tziboula A, McNulty D, et al. Milk protein-carrageenan interactions. Food Hydrocoll. 1997;11(1):101–107.

[53] Fahoum L, Moscovici A, David S, et al. Digestive fate of dietary carrageenan: evidence of interference with digestive proteolysis and disruption of gut epithelial function. Mol Nut Food Res. 2017;61(3):1600545. 10.1002/mnfr.201600545 27718308

[54] Bhattacharyya S, Gill R, Mei LC, et al. Toll-like receptor 4 mediates induction of the Bcl10-NFκB- interleukin-8 inflammatory pathway by carrageenan in human intestinal epithelial cells. J Biol Chem. 2008;283(16):10550–10558. 18252714 10.1074/jbc.M708833200 PMC2447641

[55] Borthakur A, Bhattacharyya S, Anbazhagan AN, et al. Prolongation of carrageenan-induced inflammation in human colonic epithelial cells by activation of an NFκB-BCL10 loop. Biochim Biophys Acta, Mol Basis Dis. 2012;1822(8):1300–1307. 10.1016/j.bbadis.2012.05.001 PMC3656608 22579587

[56] Benard C, Cultrone A, Michel C, et al. Degraded carrageenan causing colitis in rats induces TNF secretion and ICAM-1 upregulation in monocytes through NF-κB activation. PLoS ONE. 2010;5(1):e8666. . 20072622 10.1371/journal.pone.0008666 PMC2800179

[57] Gibson GR, Macfarlane S, Cummings JH. The fermentability of polysaccharides by mixed human faecal bacteria in relation to their suitability as bulk-forming laxatives. Lett Appl Microbiol. 1990;11(5):251–254.

[58] Michel C, Macfarlane GT. Digestive fates of soluble polysaccharides from marine macroalgae: involvement of the colonic microflora and physiological consequences for the host. J Appl Bacteriol. 1996;80(4):349–369. 8849638 10.1111/j.1365-2672.1996.tb03230.x

[59] Miller IJ, Blunt JW. Desulfation of algal galactans. Carbohydr Res. 1998;309(1):39–43.

[60] Sun Y, Cui X, Duan M, et al. *In vitro* fermentation of κ-carrageenan oligosaccharides by human gut microbiota and its inflammatory effect on HT29 cells. J Funct Foods. 2019;59:80–91.

[61] Gao Y, Lv X, Yang H, et al. Isoliquiritigenin exerts antioxidative and anti-inflammatory effects via activating the KEAP-1/Nrf2 pathway and inhibiting the NF-κB and NLRP3 pathways in carrageenan-induced pleurisy. Food Function. 2020;11(3):2522–2534. 32141447 10.1039/c9fo01984g

[62] Mi Y, Chin YX, Cao WX, et al. Native κ-carrageenan induced-colitis is related to host intestinal microecology. Int J Biol Macromol. 2020;147:284–294. 31926226 10.1016/j.ijbiomac.2020.01.072

[63] Ou Z, Zhao J, Zhu L, et al. Anti-inflammatory effect and potential mechanism of betulinic acid on λ-carrageenan-induced paw edema in mice. Biomed Pharmacother. 2019;118:109347. 31545273 10.1016/j.biopha.2019.109347

[64] Sur B, Kang S, Kim M, et al. Inhibition of carrageenan/kaolin-induced arthritis in rats and of inflammatory cytokine expressions in human IL-1β-stimulated fibroblast-like synoviocytes by a benzylideneacetophenone derivative. Inflammation. 2019;42(3):928–936. 30565030 10.1007/s10753-018-0947-8 PMC6527524

[65] Guo J, Han S, Lu X, et al. κ-Carrageenan hexamer have significant anti-inflammatory activity and protect RAW264.7 Macrophages by inhibiting CD14. J Funct Foods. 2019;57:335–344.

[66] Lee D, Swan CK, Suskind D, et al. Children with Crohn’s disease frequently consume select food additives. Dig Dis Sci. 2018;63(10):2722–2728. . 29862484 10.1007/s10620-018-5145-x PMC6290903

[67] duPreez R, Paul N, Mouatt P, et al. Carrageenans from the red seaweed *Sarconema filiforme* attenuate symptoms of diet-induced metabolic syndrome in rats. Mar Drugs. 2020;18(2):97. 10.3390/md18020097 PMC7073600 32023936

[68] Klisch S, Dicaprio E, Soule KE, et al. Safety of carrageenan. University of California, Agriculture and Natural Resources; 2018. Davis.

[69] Armisén R, Galatas F Agar. In: Phillips GO, Williams PA, editors. Handbook of Hydrocolloids: second Edition. Cambridge: Elsevier Inc; 2009. p. 82–107.

[70] Imeson A Agar. In: Alan I, editor. Food Stabilisers, Thickeners and Gelling Agents. Hoboken: Wiley Online Library; 2009a. p. 31–49.

[71] Selby HH, Whistler RL Agar. In: BeMiller J, Whistler R, editors. Industrial Gums: polysaccharides and Their Derivatives: third Edition. Cambridge: Elsevier Inc; 2012. p. 87–103.

[72] Mostafavi FS, Zaeim D. Agar-based edible films for food packaging applications - A review. Int J Biol Macromol. 2020;159:1165–1176. 32442572 10.1016/j.ijbiomac.2020.05.123

[73] Ouyang QQ, Hu Z, Li SD, et al. Thermal degradation of agar: mechanism and toxicity of products. Food Chem. 2018;264:277–283. 29853377 10.1016/j.foodchem.2018.04.098

[74] Ellis AL, Norton AB, Mills TB, et al. Stabilisation of foams by agar gel particles. Food Hydrocoll. 2017;73:222–228.

[75] Helgerud T, Gaserød O, Fjæreide T, et al. Alginates. In: Alan I, editor. Food Stabilisers, Thickeners and Gelling Agents. Hoboken: Wiley Online LibraryP. 50–72; 2010.

[76] Draget KI Alginates. In: Phillips, G, Williams, P, editors. Handbook of Hydrocolloids: second Edition. Cambridge: Elsevier Inc; 2009. p. 807–828.

[77] Paredes Juárez GA, Spasojevic M, Faas MM, et al. Immunological and technical considerations in application of alginate-based microencapsulation systems. Front Bioeng Biotechnol. 2014;2(26):1–15. 25147785 10.3389/fbioe.2014.00026 PMC4123607

[78] Aarstad O, Heggset EB, Pedersen IS, et al. Mechanical properties of composite hydrogels of alginate and cellulose nanofibrils. Polymers. 2017;9(8):378. 10.3390/polym9080378 PMC6418825 30971055

[79] Xu X, Han Q, Shi J, et al. Structural, thermal and rheological characterization of bovine serum albumin binding with sodium alginate. J Mol Liq. 2020;299:112123.

[80] Li Y, Dou X, Pang J, et al. Improvement of fucoxanthin oral efficacy via vehicles based on gum Arabic, gelatin and alginate hydrogel: delivery system for oral efficacy enhancement of functional food ingredients. J Funct Foods. 2019;63:103573.

[81] Mahmoud M, Abdallah NA, El-Shafei K, et al. Survivability of alginate-microencapsulated *Lactobacillus plantarum* during storage, simulated food processing and gastrointestinal conditions. Heliyon. 2020;6(3):e03541. 32190759 10.1016/j.heliyon.2020.e03541 PMC7068628

[82] Dehkordi SS, Alemzadeh I, Vaziri AS, et al. Optimization of alginate-whey protein isolate microcapsules for survivability and release behavior of probiotic bacteria. Appl Biochem Biotechnol. 2020;190(1):182–196. 31313242 10.1007/s12010-019-03071-5

[83] Bambace MF, Alvarez MV, Moreira M, et al. Novel functional blueberries: fructo-oligosaccharides and probiotic lactobacilli incorporated into alginate edible coatings. Food Res Int. 2019;122:653–660. 31229124 10.1016/j.foodres.2019.01.040

[84] Su C, Feng Y, Ye J, et al. Effect of sodium alginate on the stability of natural soybean oil body emulsions. RSC Adv. 2018;8(9):4731–4741. . 10.1039/c7ra09375f PMC9077793 35539521

[85] Chen J, Mu T, Goffin D, et al. Application of soy protein isolate and hydrocolloids based mixtures as promising food material in 3D food printing. J Food Eng. 2019;261:76–86.

[86] Jungst T, Smolan W, Schacht K, et al. Strategies and molecular design criteria for 3D printable hydrogels. Chem Rev. 2016;116(3):1496–1539. 26492834 10.1021/acs.chemrev.5b00303

[87] Hinton TJ, Jallerat Q, Palchesko RN, et al. Three-dimensional printing of complex biological structures by freeform reversible embedding of suspended hydrogels. Sci Adv. 2015;1(9):e1500758. . 26601312 10.1126/sciadv.1500758 PMC4646826

[88] Jia J, Richards DJ, Pollard S, et al. Engineering alginate as bioink for bioprinting. Acta Biomater. 2014;10(10):4323–4331. . 24998183 10.1016/j.actbio.2014.06.034 PMC4350909

[89] Corstens MN, Troost FJ, Alleleyn AME, et al. Encapsulation of lipids as emulsion-alginate beads reduces food intake: a randomized placebo-controlled cross-over human trial in overweight adults. Nutr Res. 2019;63:86–94. 30824401 10.1016/j.nutres.2018.12.004

[90] Asnani GP, Bahekar J, Kokare CR. Development of novel pH–responsive dual crosslinked hydrogel beads based on *Portulaca oleracea* polysaccharide-alginate-borax for colon specific delivery of 5-fluorouracil. J Drug Delivery Sci Technol. 2018;48:200–208.

[91] Chenoweth MB. The toxicity of sodium alginate in cats. Ann Surg. 1948;127(6):1173–1181. 17859157 10.1097/00000658-194806000-00006 PMC1513692

[92] Solandt OM. Some observations upon sodium alginate. Quarterly Journal of Experimental Physiology and Cognate Medical Sciences. 1941;31(1):25–30.

[93] Yang D, Jones KS. Effect of alginate on innate immune activation of macrophages. Journal of Biomedical Materials Research - Part A. 2009;90(2):411–418. 18523947 10.1002/jbm.a.32096

[94] Kim AR, Hwang JH, Kim HM, et al. Reduction of inflammatory reaction in the use of purified alginate microcapsules. J Biomater Sci Polym Ed. 2013;24(9):1084–1098. . 23683040 10.1080/09205063.2012.735100

[95] Wang Y, Li L, Ye C, et al. Alginate oligosaccharide improves lipid metabolism and inflammation by modulating gut microbiota in high-fat diet fed mice. Appl Microbiol Biotechnol. 2020;104(8):3541–3554. 32103315 10.1007/s00253-020-10449-7

[96] Shteyer E, BenYa’acov A, Zolotaryova L, et al. Prevention of acetaminophen-induced liver injury by alginate. Toxicol Appl Pharmacol. 2019;363:72–78. 30468816 10.1016/j.taap.2018.11.008

[97] Qureshi D, Nayak SK, Maji S, et al. Carrageenan: a wonder polymer from marine algae for potential drug delivery applications. Curr Pharm Des. 2019;25(11):1172–1186. 31465278 10.2174/1381612825666190425190754

[98] Sedayu BB, Cran MJ, Bigger SW. A review of property enhancement techniques for carrageenan-based films and coatings. Carbohydr Polym. 2019;216:287–302. 31047069 10.1016/j.carbpol.2019.04.021

[99] Gioele C, Marilena S, Valbona A, et al. *Gracilaria gracilis*, source of agar: a short review. Curr Org Chem. 2017;21(5):380–386.

[100] Lee W-K, Lim -Y-Y, Leow AT-C, et al. Biosynthesis of agar in red seaweeds: a review. Carbohydr Polym. 2017;164:23–30. 28325321 10.1016/j.carbpol.2017.01.078

[101] Sansonetti A, Bertasa M, Canevali C, et al. A review in using agar gels for cleaning art surfaces. J CultHeritage. 2020;44:285–296.

[102] Wang B, Wan Y, Zheng Y, et al. Alginate-based composites for environmental applications: a critical review. Crit Rev Environ Sci Technol. 2019;49(4):318–356. . 10.1080/10643389.2018.1547621 PMC8193857 34121831

[103] Agüero L, Zaldivar-Silva D, Peña L, et al. Alginate microparticles as oral colon drug delivery device: a review. Carbohydr Polym. 2017;168:32–43. 28457455 10.1016/j.carbpol.2017.03.033

[104] Reakasame S, Boccaccini AR. Oxidized alginate-based hydrogels for tissue engineering applications: a review. Biomacromolecules. 2018;19(1):3–21. 29172448 10.1021/acs.biomac.7b01331

[105] Varaprasad K, Jayaramudu T, Kanikireddy V, et al. Alginate-based composite materials for wound dressing application: a mini review. Carbohydr Polym. 2020;236:116025. 32172843 10.1016/j.carbpol.2020.116025

[106] McKim JM, Baas H, Rice GP, et al. Effects of carrageenan on cell permeability, cytotoxicity, and cytokine gene expression in human intestinal and hepatic cell lines. Food Chem Toxicol. 2016;96:1–10. 27424122 10.1016/j.fct.2016.07.006

[107] Tobacman JK. Reply to comments regarding “The Carrageenan Controversy.”. J Appl Phycol. 2017;29(5):2209–2211.

[108] Weiner ML, McKim JM. Comment on “Revisiting the carrageenan controversy: do we really understand the digestive fate and safety of carrageenan in our foods?” by S. David, C. S. Levi, L. Fahoum, Y. Ungar, E. G. Meyron-Holtz, A. Shpigelman and U. Lesmes,: food Funct., 2018, 9, 1. Food Function. 2019;10(3):1760–1762. 30794268 10.1039/c8fo01282b

[109] David S, Fahoum L, Rozen G, et al. Reply to the Comment on “Revisiting the carrageenan controversy: do we really understand the digestive fate and safety of carrageenan in our foods?” by M. Weiner and J. McKim, Food Funct. 2019 Food Function. 2019;10 1:1763–1766. 30794278 10.1039/c9fo00018f

[110] Beetul K, Gopeechund A, Kaullysing D, et al. Challenges and opportunities in the present era of marine algal applications. In: Thajuddin, N, Dhanasekaran, D, editors. Algae - Organisms for Imminent Biotechnology. London: InTech; 2016. p. 237–276.

[111] Enamala MK, Enamala S, Chavali M, et al. Production of biofuels from microalgae - A review on cultivation, harvesting, lipid extraction, and numerous applications of microalgae. *Renewable and Sustainable Energy Reviews*. 2018; 94: 49–68.

[112] Villanueva RD, Mendoza WG, Rodrigueza MRC, et al. Structure and functional performance of gigartinacean kappa-iota hybrid carrageenan and solieriacean kappa-iota carrageenan blends. Food Hydrocoll. 2004;18(2):283–292.

[113] Bertasa M, Botteon A, Brambilla L, et al. Cleaning materials: a compositional multi-analytical characterization of commercial agar powders. J Anal Appl Pyrolysis. 2017;125:310–317.

